# The impact of high-performance work systems on employees’ subjective well-being: the mediating effect of emotional commitment and the moderating effect of interpersonal fairness

**DOI:** 10.3389/fpsyg.2026.1807123

**Published:** 2026-08-14

**Authors:** Rongcheng Liang

**Affiliations:** 1 Department of Emergency Management Training, Party School of the Communist Party of China Shandong Provincial Committee (Shandong Academy of Governance), Jinan, China; 2 School of International Relations and Public Affairs, Fudan University, Shanghai, China

**Keywords:** emotional commitment, high-performance work systems, interpersonal fairness, self-determination theory, subjective well-being

## Abstract

Employee well-being has become a strategic imperative as organizations worldwide confront rising burnout, disengagement, and the human costs of intensified competition. Yet organizations face a paradox: the high-performance work systems (HPWS) they adopt to remain competitive may either nourish or erode the well-being of the employees who enact them. Motivated by this tension, this study investigates not only whether HPWS influences employees’ subjective well-being (SWB), but also how and when this influence occurs. Drawing on self-determination theory, it proposes that HPWS enhances SWB by satisfying employees’ basic psychological needs for autonomy, competence, and relatedness, thereby strengthening their emotional commitment to the organization, and that interpersonal fairness – the need-supportive quality of day-to-day interpersonal treatment – conditions this process. Three-wave, multi-source data were collected from 412 employees across 68 teams in Chinese technology companies, and the hypothesized moderated mediation model was tested using hierarchical linear modeling and bootstrap methods. The results revealed that: (1) HPWS positively influences employees’ SWB; (2) emotional commitment partially mediates this relationship; (3) interpersonal fairness moderates the HPWS-emotional commitment link, such that the positive effect is stronger when interpersonal fairness is high; and (4) the indirect effect of HPWS on SWB through emotional commitment is likewise stronger when interpersonal fairness is high. These findings extend self-determination theory into the strategic human resource management domain by identifying emotional commitment as a need-based transmission mechanism and interpersonal fairness as a need-supportive boundary condition, and they offer actionable guidance for designing HPWS that pursue performance goals without sacrificing employee well-being. By responding to recent calls for mechanism-focused and context-sensitive HRM-well-being research, the study shows how performance systems can be designed to serve both organizational and employee interests.

## 1 Introduction

Employee well-being has moved from the periphery of management concern to the center of organizational strategy and public policy. Accumulating evidence shows that employees’ subjective well-being (SWB) – their cognitive and affective evaluations of their lives (Diener et al., 2018) – predicts job performance, creativity, retention, and customer satisfaction, whereas its absence manifests in burnout, disengagement, and escalating health-care costs (Peccei and Van De Voorde, 2019; Gagne et al., 2022). The COVID-19 pandemic and the subsequent re-evaluation of work have further amplified these stakes, pushing organizations to treat well-being not as a discretionary benefit but as a core component of sustainable competitive advantage (Kooij et al., 2013; Gagne et al., 2022). Understanding which managerial practices foster – or undermine – employee well-being has therefore become one of the most consequential questions in contemporary human resource management. Recent systematic reviews confirm that employee well-being has become a central criterion in HRM research and that HPWS-type practice bundles are among its most frequently examined antecedents (Bhoir and Sinha, 2024; Kaur and Malik, 2025).

High-performance work systems (HPWS) – bundles of human resource practices such as selective staffing, extensive training, developmental performance management, competitive compensation, employee involvement, information sharing, and job security (Combs et al., 2006; Boxall and Macky, 2009; Jiang et al., 2012) – sit at the heart of this question. Although the performance-enhancing effects of HPWS are well documented (Combs et al., 2006; Jiang et al., 2012), their consequences for employee well-being remain one of the most polarized debates in the field. The mutual gains perspective holds that HPWS benefits both organizations and employees by enriching jobs and investing in people (Guest, 2017; Peccei and Van De Voorde, 2019); the conflicting outcomes perspective counters that HPWS intensifies work, elevates strain, and sacrifices well-being for performance (Guest, 2017; Katou, 2022). Two decades of research have not resolved this tension, and recent reviews conclude that the field still lacks a coherent account of when and why HPWS helps or harms employees (Peccei and Van De Voorde, 2019; Kooij et al., 2013; Li et al., 2024).

We argue that this impasse persists because the debate has been framed at the wrong level of analysis. Rather than asking whether HPWS affects well-being – a question that treats HPWS as a monolithic treatment and employees as passive recipients – the more productive questions are how HPWS is psychologically transformed into well-being and under what interactional conditions this transformation succeeds or fails. Three specific gaps follow. First, the psychological mechanism remains a black box: existing studies catalog practices and correlate them with outcomes but rarely explain how employees cognitively and affectively interpret HPWS, leaving the mediating process under-theorized (Peccei and Van De Voorde, 2019; Van De Voorde and Beijer, 2015). Second, the interactional context in which HPWS is enacted has been neglected: identical practices can be delivered with dignity and respect or with indifference and disrespect, yet boundary conditions rooted in the quality of interpersonal treatment have received little systematic attention (Colquitt et al., 2013). Third, the evidence base is dominated by Western, single-level, cross-sectional designs; the multi-level psychological processes through which HPWS operates in non-Western, high-intensity employment contexts – such as China’s technology sector, where performance pressure and employee well-being coexist in visible tension – remain largely untested (Katou, 2022). Recent studies continue to document these blind spots: HPWS has been linked to satisfaction and performance without modeling the psychological transmission mechanism (Dorta-Afonso et al., 2023; Han et al., 2023), and evidence from China’s high-intensity technology sector remains scarce (Li and Rasiah, 2025).

To address these gaps, we build our model on a single, integrative theoretical lens: self-determination theory (SDT; Deci et al., 2017; Ryan, 2023). SDT is particularly suited to this task for three reasons. First, it specifies a concrete psychological mechanism: social contexts affect well-being to the extent that they satisfy or thwart the basic psychological needs for autonomy, competence, and relatedness (Van den Broeck et al., 2016). Second, it explains attachment: when the work environment supports these needs, employees internalize organizational goals and values, developing the affective bond captured by emotional commitment (Gagne and Deci, 2005; Meyer and Maltin, 2010). Third, it provides a principled account of moderation: the same managerial practices can be experienced as need-supportive or as controlling depending on the quality of the interpersonal climate, which allows SDT to reconcile the mutual gains and conflicting outcomes perspectives rather than merely arbitrating between them (Deci et al., 2017; Gagne et al., 2022). Using one theory throughout – rather than accumulating several – yields a parsimonious chain of reasoning in which every hypothesis is derived from the same motivational logic.

Therefore, this study explores the influence of high-performance work systems on employees’ subjective well-being, as well as the mediating role of emotional commitment and the moderating role of interpersonal fairness. The study makes three contributions: First, it reframes the HPWS-well-being debate in need-based terms: HPWS is not inherently beneficial or harmful; its effect depends on whether its practices are experienced as satisfying or thwarting basic psychological needs, a reframing that converts a stalled controversy into a testable set of mechanism-focused questions. Second, by theorizing emotional commitment as an internalization-based transmission mechanism, it opens the black box between HPWS and well-being and explains employees’ cognitive and affective interpretation of HR practices – an interpretive process that prior research has described but not explained (Van De Voorde and Beijer, 2015). Third, by specifying interpersonal fairness as an enabling boundary condition and testing the full moderated mediation chain in a Chinese technology context, it extends SDT’s contextual reach and identifies the interactional conditions under which HPWS realizes its well-being potential.

## 2 Theory and hypotheses development

### 2.1 Self-determination theory

Self-determination theory is a macro-theory of human motivation, development, and wellness that distinguishes the quality of motivation from its quantity (Deci and Ryan, 2000). Its core proposition is that all human beings possess three basic psychological needs: autonomy (experiencing one’s actions as self-endorsed and volitional), competence (feeling effective and capable of mastering challenges), and relatedness (feeling connected to, and cared about by, others). When the social environment supports these needs, individuals display autonomous motivation, optimal functioning, and psychological well-being; when the environment thwarts these needs – through control, pressure, or relational neglect – motivation becomes controlled and well-being deteriorates, even if performance is temporarily sustained (Deci et al., 2017; Ryan and Deci, 2020; Van den Broeck et al., 2016).

Two further propositions of SDT are central to our model. The first concerns internalization: in need-supportive contexts, people actively assimilate external values and regulations, transforming what the organization wants into what they personally endorse (Gagne and Deci, 2005). In work organizations, internalization manifests as affective attachment to, and identification with, the organization – the construct captured by emotional commitment (Meyer and Allen, 1991; Meyer and Maltin, 2010). The second concerns the interpersonal climate: supervisors and organizational authorities constitute the most immediate social context of work, and their interpersonal style – supportive versus controlling, respectful versus dismissive – determines whether managerial practices are experienced as need-satisfying or need-thwarting (Deci et al., 2017; Gagne et al., 2022). SDT thus offers a unified account of the mechanism (need satisfaction and internalization), the mediator (emotional commitment), and the boundary condition (the need-supportive quality of interpersonal treatment) in the HPWS-well-being relationship.

Adopting SDT also responds to a substantive weakness in the HPWS literature. Much existing research is descriptive: it demonstrates covariation between HR practices and well-being outcomes without explaining the psychological process that produces it, and it often assembles theoretical narratives post hoc from several loosely connected frameworks (Peccei and Van De Voorde, 2019). Attribution research has shown that employees’ well-being reactions depend on how they interpret management’s intentions behind HR practices (Van De Voorde and Beijer, 2015), yet this stream stops short of specifying the motivational mechanism through which such interpretations form and translate into well-being. Re-examining the HPWS-well-being relationship is therefore not a redundant retest of an established association; it is an attempt to explain an association whose generative mechanism and boundary conditions remain unknown, in a context – Chinese technology firms – where the tension between performance demands and employee well-being is unusually sharp.

### 2.2 High-performance work systems and employee subjective well-being

Viewed through SDT, the constituent practices of HPWS map directly onto the three basic psychological needs. Extensive training, developmental performance management, and career development opportunities build mastery and satisfy the need for competence; employee involvement, participative decision-making, and information sharing grant voice and ownership, satisfying the need for autonomy; and team-based work design, job security, and collective rewards signal membership and care, satisfying the need for relatedness (Deci et al., 2017; Van den Broeck et al., 2016; Gagne et al., 2022). Because the satisfaction of these needs is the proximal determinant of wellness, employees working under well-developed HPWS should experience higher vitality, more positive affect, and greater satisfaction with their lives. Consistent with this mapping, recent work grounded in the ability-motivation-opportunity framework shows that HPWS-type bundles enhance employee attitudes and well-being across diverse sectors (Bos-Nehles et al., 2023; Padamata and Vangapandu, 2024).

This need-based account also clarifies why the dark-side critique, though important, does not imply a negative main effect. Critics rightly observe that HPWS can be enacted in ways that intensify demands and pressure (Guest, 2017; Katou, 2022; Demerouti and Bakker, 2023). SDT shows, however, that strain is not an intrinsic property of HR practices but a consequence of controlling enactment: the same performance management system can be experienced as an opportunity for growth or as an instrument of surveillance, depending on how it is interpersonally delivered (Deci et al., 2017). Practices designed around investment, involvement, and security are, on average, more need-supportive than need-thwarting, which explains why meta-analytic and review evidence finds positive net associations between HPWS and employee well-being (Jiang et al., 2012; Peccei and Van De Voorde, 2019). We therefore expect a positive main effect, while explicitly modeling the interpersonal condition that governs when this effect is realized (Hypothesis 3).

Hypothesis 1: High-performance work systems positively influence employees’ subjective well-being.

### 2.3 The mediating role of emotional commitment

The first stage of the mediating mechanism is grounded in SDT’s internalization proposition. When HPWS practices satisfy employees’ needs for autonomy, competence, and relatedness, employees do not merely comply with organizational expectations; they come to endorse organizational goals and values as their own (Gagne and Deci, 2005; Deci et al., 2017). This internalization process is experienced affectively as attachment, identification, and a sense of belonging – precisely the content of emotional commitment (Meyer and Allen, 1991). Commitment research supports this reading: affective bonds to the organization arise from experiences of support, fairness, and growth rather than from instrumental calculation (Meyer and Maltin, 2010), and HPWS practices such as developmental opportunities and participative management have been linked empirically to stronger affective attachment (Sun et al., 2007; Takeuchi et al., 2009). Recent cross-sector evidence corroborates this first stage: HPWS has been shown to build affective commitment both directly and through need-based experiences such as thriving at work (Alothmany et al., 2023).

The second stage links emotional commitment to subjective well-being. SDT holds that acting from internalized, self-congruent motives is itself a source of wellness: employees who are emotionally committed experience their work role as an expression of who they are rather than as an externally imposed obligation (Meyer and Maltin, 2010). Such employees derive meaning, identity, and secure belonging from their organizational membership, and these relational resources spill over into global life evaluations, raising life satisfaction and positive affect (Meyer and Maltin, 2010; Diener et al., 2018). Emotional commitment thus constitutes the psychological pathway – need satisfaction, internalization, attachment, well-being – through which the structural properties of HPWS are converted into subjective well-being. Specifying this pathway answers calls to open the black box between HRM and well-being with an explicit, theory-derived mechanism (Peccei and Van De Voorde, 2019). Recent evidence reinforces this second stage as well: affective commitment transmits the effects of high-performance work practices onto valued organizational outcomes, confirming its role as a transmission channel rather than a mere correlate (Rawashdeh et al., 2025).

Hypothesis 2: Emotional commitment mediates the relationship between high-performance work systems and employees’ subjective well-being.

### 2.4 The moderating role of interpersonal fairness

SDT assigns the interpersonal climate a decisive role in need satisfaction: because supervisors are the most salient representatives of the organization, the way they treat employees during the enactment of HR practices determines whether those practices are experienced as supportive or controlling (Deci et al., 2017; Gagne et al., 2022). Interpersonal fairness – treatment with dignity, respect, and politeness (Bies and Moag, 1986) – is the justice dimension that captures exactly this quality of treatment (Colquitt, 2001; Colquitt et al., 2013). Respectful treatment directly nourishes the need for relatedness and signals that performance expectations are invitations rather than impositions, protecting felt autonomy; disrespectful treatment does the opposite, recasting developmental feedback as evaluation and involvement as extraction (Deci et al., 2017).

We therefore conceptualize the moderation as enhancing (enabling) rather than buffering. Interpersonal fairness does not protect employees against an inherently harmful treatment; it unlocks the need-satisfying potential of a beneficial one. When interpersonal fairness is high, HPWS practices are delivered in a need-supportive climate, internalization proceeds, and the HPWS-commitment link is strong; when fairness is low, the same practices are experienced as controlling, internalization is blocked, and the link weakens. This framing differs from resource- or exchange-based accounts, which treat fair treatment as an additional resource or as a trigger of reciprocity; in SDT terms, fairness is a condition of the motivational process itself. Meta-analytic evidence that interactional justice shapes employee responses to managerial action is consistent with this reasoning (Colquitt et al., 2013). Recent moderated models echo this enabling logic: cultural and relational conditions, such as power distance orientation and hierarchy culture, determine whether HPWS translates into favorable employee outcomes (Li and Rasiah, 2025; Rawashdeh et al., 2025).

Hypothesis 3: Interpersonal fairness moderates the relationship between high-performance work systems and emotional commitment, such that the positive relationship is stronger when interpersonal fairness is high.

### 2.5 The moderated mediation model

Combining Hypotheses 2 and 3 yields a moderated mediation model in which interpersonal fairness conditions the indirect effect of HPWS on subjective well-being through emotional commitment (Preacher et al., 2007; Edwards and Lambert, 2007; Hayes, 2015). This hypothesis has an independent theoretical rationale rather than being a statistical extension of Hypotheses 2 and 3. Within SDT, internalization is not a one-off event at the first stage of a causal chain but a continuing motivational process: the affective bond formed through need satisfaction sustains well-being only while the surrounding climate remains need-supportive (Deci et al., 2017). A disrespectful climate therefore attenuates the entire HPWS to emotional commitment to well-being sequence, because it simultaneously blocks the formation of internalized attachment and corrodes the wellness value of whatever attachment already exists. Conversely, a respectful climate amplifies the whole sequence: need-supportive enactment strengthens commitment, and the resulting attachment is experienced as genuine rather than instrumental, maximizing its contribution to life evaluations. Methodologically, such first-stage moderated mediation is evaluated through the index of moderated mediation with bootstrap confidence intervals (Hayes, 2018).

**FIGURE 1:**
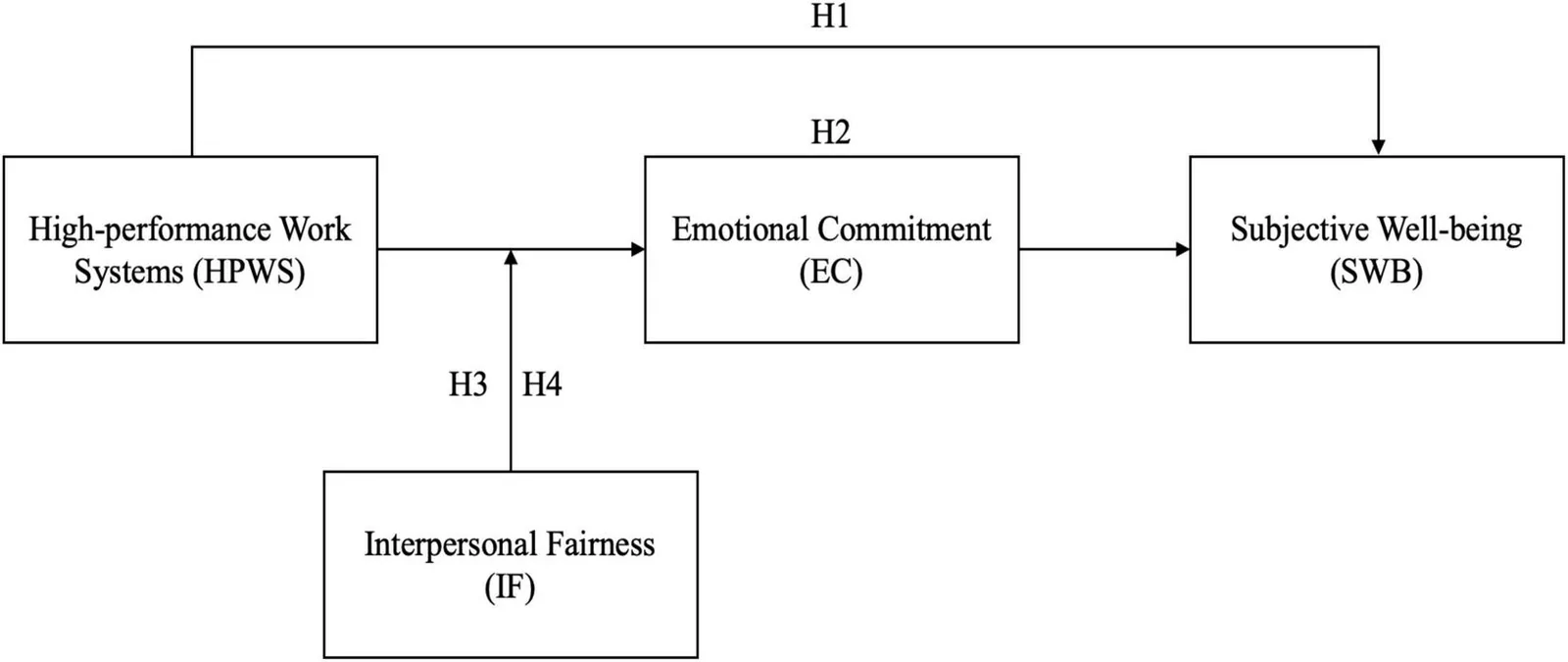
Research framework.

Hypothesis 4: Interpersonal fairness moderates the indirect relationship between high-performance work systems and subjective well-being through emotional commitment, such that the indirect effect is stronger when interpersonal fairness is high. Figure 1 illustrates the research framework of this study.

## 3 Materials and methods

### 3.1 Research context

We situated the study in China’s technology sector – specifically firms in Beijing, Shanghai, and Shenzhen – for theoretical as well as practical reasons. The sector combines exceptionally intensive performance management with fierce competition for scarce digital talent, making HPWS both widespread and consequential for employee well-being (Li and Rasiah, 2025). The context is also marked by a visible societal tension: the prevalence of extended work schedules popularly termed 996 (9 a.m. to 9 p.m., 6 days a week) has triggered public debate and employee protests and, in 2021, joint guidance from China’s Supreme People’s Court and Ministry of Human Resources and Social Security clarifying that such schedules violate statutory working-hour limits (Supreme People’s Court and Ministry of Human Resources and Social Security of the People’s Republic of China, 2021). Meanwhile, the sector’s young, highly educated workforce increasingly evaluates employers by their contribution to personal well-being. These conditions create a theoretically informative setting: HPWS is implemented under high performance pressure, so the need-supportive versus controlling quality of its enactment varies meaningfully across teams, providing the variance required to test our moderation logic. Anchoring the study in this national-industrial context also allows it to contribute meso-level, context-specific theory rather than assuming the universal portability of Western findings (Homer and Lim, 2024).

### 3.2 Sample and procedure

The data for this study were collected from technology companies in Beijing, Shanghai, and Shenzhen, China. We employed a multi-source, time-lagged research design to minimize common method bias (Podsakoff et al., 2003). Data were collected in three waves, with a 4-week interval between each wave. In the first wave (Time 1), team leaders reported on the HPWS practices implemented in their teams. In the second wave (Time 2), employees reported their perceptions of interpersonal fairness and their emotional commitment to the organization. In the third wave (Time 3), employees reported their subjective well-being. This time-lagged design allows for stronger causal inferences by establishing temporal precedence among the study variables.

Participants were recruited through personal contacts and professional networks, which we used solely to gain organizational access. Several steps were taken to mitigate the sampling bias that such recruitment may introduce. First, the personal contacts played no role in selecting teams or individual respondents; once a company agreed to participate, its HR department provided a full team roster, from which we randomly selected teams, and all members of the selected teams were invited to participate. Second, the participating companies varied in size, ownership structure, and technology subsector, reducing the risk that the findings reflect a single organizational culture. Third, participation was voluntary, employees completed the online surveys outside working hours using unique identification codes, and no employer representative had any involvement in data collection or access to individual responses, which limits both coercion and social desirability bias. Fourth, we assessed potential non-response and attrition bias by comparing early and late respondents, and completers versus dropouts, on all demographic variables and Time 1 measures; none of the comparisons was statistically significant, suggesting that self-selection did not materially distort the sample. All participants were informed that their responses would be used for research purposes only and would not be shared with their employers. These procedural safeguards follow current methodological recommendations for minimizing common method bias at the design stage (Podsakoff et al., 2024).

A total of 520 employees from 85 teams were invited to participate in the study. After excluding incomplete responses and participants who did not complete all three waves of data collection, the final sample consisted of 412 employees from 68 teams, representing a response rate of 79.2%. The average team size was 6.06 members (SD = 2.34). Among the participants, 54.4% were male, and the average age was 31.2 years (SD = 5.8). The average organizational tenure was 4.3 years (SD = 3.1), and 62.1% held bachelor’s degrees or higher. The demographic characteristics of our sample are consistent with the technology sector workforce in China, which tends to be relatively young and well-educated.

### 3.3 Measures

All measures used in this study were originally developed in English and translated into Chinese following the standard back-translation procedure to ensure equivalence across languages. Unless otherwise noted, all items were measured on a 7-point Likert scale ranging from 1 (strongly disagree) to 7 (strongly agree).

High-Performance Work Systems. HPWS was measured using a 24-item scale adapted from Sun et al. (2007) and Takeuchi et al. (2009). The scale assesses seven dimensions of HPWS: selective hiring (3 items), extensive training (4 items), developmental performance management (3 items), competitive compensation (3 items), employee involvement (4 items), information sharing (4 items), and job security (3 items). Sample items include ‘Our company uses rigorous selection procedures to hire the best candidates’ and ‘Our company provides extensive training programs to enhance employees’ skills.’ Team leaders rated the extent to which each practice was implemented in their teams. The Cronbach’s alpha for this scale was 0.92.

Emotional Commitment. Emotional commitment was measured using the 6-item scale developed by Meyer and Allen (1991). Sample items include ‘I would be very happy to spend the rest of my career with this organization’ and ‘I really feel as if this organization’s problems are my own.’ The Cronbach’s alpha for this scale was 0.89. Throughout this article, ‘emotional commitment’ denotes the affective component of organizational commitment – emotional attachment to, identification with, and involvement in the organization (Meyer and Allen, 1991) – and is used interchangeably with ‘affective commitment’ in the commitment literature.

Interpersonal Fairness. Interpersonal fairness was measured using the 4-item scale developed by Colquitt (2001) based on the work of Bies and Moag (1986). This scale assesses the extent to which employees are treated with dignity, respect, and politeness by organizational authorities. Sample items include ‘My supervisor treats me with dignity’ and ‘My supervisor treats me with respect.’ The Cronbach’s alpha for this scale was 0.91.

Subjective Well-being. Subjective well-being was measured using the 5-item Satisfaction with Life Scale (SWLS) developed by Diener et al. (1985). Sample items include ‘In most ways my life is close to my ideal’ and ‘I am satisfied with my life.’ The Cronbach’s alpha for this scale was 0.88.

Control Variables. We controlled for gender (0 = female, 1 = male), age (in years), education level (1 = high school or below, 2 = associate degree, 3 = bachelor’s degree, 4 = master’s degree or above), organizational tenure (in years), and team size as a team-level variable that may affect the implementation and effectiveness of HPWS.

### 3.4 Data analysis

We used hierarchical linear modeling (HLM) to test our hypotheses because the data have a nested structure with employees (Level 1) nested within teams (Level 2). HLM appropriately accounts for the non-independence of observations within teams and allows for the simultaneous examination of within-team and between-team effects. The appropriateness of this multilevel specification was confirmed: intraclass correlations indicated that a substantial proportion of the variance in emotional commitment and subjective well-being resided between teams (both ICC (1) values exceeded the conventional 0.05 threshold). All continuous predictors were grand-mean centered before computing the interaction term.

To test the mediating effect of emotional commitment (Hypothesis 2), we conducted a multilevel mediation analysis using the Monte Carlo method for assessing mediation with 20,000 resamples (Preacher and Hayes, 2008). To test the moderating effect of interpersonal fairness (Hypothesis 3) and the moderated mediation effect (Hypothesis 4), we used the multilevel moderation and moderated mediation procedures outlined by Hayes (2018), including the index of moderated mediation (Hayes, 2015). We centered the predictor variables at their grand means before creating the interaction term to reduce multicollinearity and facilitate interpretation.

All analyses were conducted using Mplus 8.0 and the PROCESS macro for SPSS (Model 7 for moderated mediation). We used full information maximum likelihood (FIML) estimation to handle missing data, which provides unbiased parameter estimates under the assumption that data are missing at random.

## 4 Results

### 4.1 Confirmatory factor analysis

Before testing our hypotheses, we conducted a series of confirmatory factor analyses (CFAs) to assess the discriminant validity of the study variables. The results showed that the hypothesized four-factor model (HPWS, emotional commitment, interpersonal fairness, subjective well-being) provided a good fit to the data (Chi-square = 487.32, df = 293, CFI = 0.95, TLI = 0.94, RMSEA = 0.04, SRMR = 0.05). This model fit significantly better than a three-factor model combining emotional commitment and subjective well-being (delta Chi-square = 234.56, *p* < 0.001), a two-factor model combining all employee-reported variables (delta Chi-square = 567.89, *p* < 0.001), and a one-factor model (delta Chi-square = 892.45, *p* < 0.001), supporting the discriminant validity of the study variables. Composite reliabilities exceeded 0.80 and average variance extracted exceeded 0.50 for all four constructs, supporting convergent validity. In addition, the poor fit of the one-factor model provides a statistical check on common method bias; such checks are diagnostic rather than definitive, however, and we therefore relied primarily on the procedural remedies described in the Method section (Podsakoff et al., 2024; Howard et al., 2024).

### 4.2 Descriptive statistics and correlations

Table 1 presents the means, standard deviations, and correlations among the study variables. As expected, HPWS was positively correlated with emotional commitment (*r* = 0.42, *p* < 0.01) and subjective well-being (*r* = 0.38, *p* < 0.01). Emotional commitment was positively correlated with subjective well-being (*r* = 0.51, *p* < 0.01). Interpersonal fairness was positively correlated with emotional commitment (*r* = 0.48, *p* < 0.01) and subjective well-being (*r* = 0.44, *p* < 0.01). These correlations are consistent with our theoretical expectations and provide preliminary support for the hypothesized relationships.

**TABLE 1:** Means, standard deviations, and correlations among study variables

Variable	M	SD	1	2	3	4	5	6	7	8
1. Gender	0.54	0.50	–							
2. Age	31.20	5.80	0.08	–						
3. Education	3.12	0.85	−0.05	0.15*	–					
4. Tenure	4.30	3.10	0.03	0.42**	0.18**	–				
5. Team Size	6.06	2.34	−0.02	0.05	0.03	0.08	–			
6. HPWS	5.24	0.98	0.04	0.12*	0.15*	0.18**	0.06	–		
7. Interpersonal Fairness	5.08	1.12	−0.03	0.08	0.10	0.12*	0.04	0.45**	–	
8. Emotional Commitment	4.86	1.15	0.02	0.14*	0.12*	0.16**	0.05	0.42**	0.48**	–
9. Subjective Well-being	4.92	1.08	0.05	0.11	0.09	0.13*	0.03	0.38**	0.44**	0.51**

*N* = 412. Gender: 0 = female, 1 = male. *p < 0.05, **p < 0.01.

### 4.3 Hypothesis testing

Hypothesis 1 predicted that HPWS positively influences employees’ subjective well-being. The results from Model 1 in Table 2 show that HPWS had a significant positive effect on subjective well-being (beta = 0.38, *p* < 0.001), supporting Hypothesis 1.

**TABLE 2:** Results of hierarchical regression analysis

Variable	Model 1 (SWB)	Model 2 (EC)	Model 3 (SWB)	Model 4 (EC)	Model 5 (SWB)
Intercept	2.45** (0.32)	2.12** (0.35)	1.85** (0.31)	2.08** (0.34)	1.82** (0.30)
Control variables					
Gender	0.08 (0.09)	0.05 (0.10)	0.06 (0.08)	0.04 (0.09)	0.06 (0.08)
Age	0.02* (0.01)	0.03* (0.01)	0.01 (0.01)	0.03* (0.01)	0.01 (0.01)
Education	0.06 (0.05)	0.08 (0.06)	0.03 (0.05)	0.07 (0.05)	0.03 (0.05)
Tenure	0.04* (0.02)	0.05* (0.02)	0.02 (0.02)	0.05* (0.02)	0.02 (0.02)
Team size	−0.02 (0.03)	−0.01 (0.03)	−0.02 (0.02)	−0.01 (0.03)	−0.02 (0.02)
Main effects					
HPWS	0.38*** (0.07)	0.42*** (0.08)	0.21** (0.07)	0.40*** (0.08)	0.20** (0.07)
Interpersonal fairness				0.35*** (0.06)	0.18** (0.06)
HPWS x interpersonal Fairness				0.15** (0.05)	0.08* (0.04)
Mediator					
Emotional commitment			0.41*** (0.06)		0.40*** (0.06)
Model statistics					
R-squared	0.18	0.22	0.31	0.28	0.33
Delta R-squared	–	–	0.13	–	0.15
F	17.82***	22.45***	35.28***	28.56***	38.42***

*N* = 412. SWB = subjective well-being; EC = emotional commitment. Unstandardized regression coefficients are reported with standard errors in parentheses. *p < 0.05, **p < 0.01, ***p < 0.001.

Hypothesis 2 proposed that emotional commitment mediates the relationship between HPWS and subjective well-being. As shown in Model 2 of Table 2, HPWS had a significant positive effect on emotional commitment (beta = 0.42, *p* < 0.001). When both HPWS and emotional commitment were entered into the equation predicting subjective well-being (Model 3), the effect of HPWS decreased but remained significant (beta = 0.21, *p* < 0.01), while emotional commitment had a significant positive effect (beta = 0.41, *p* < 0.001). The bootstrap results indicated that the indirect effect of HPWS on subjective well-being through emotional commitment was significant (indirect effect = 0.17, 95% confidence interval [0.11, 0.24]), supporting Hypothesis 2.

**FIGURE 2:**
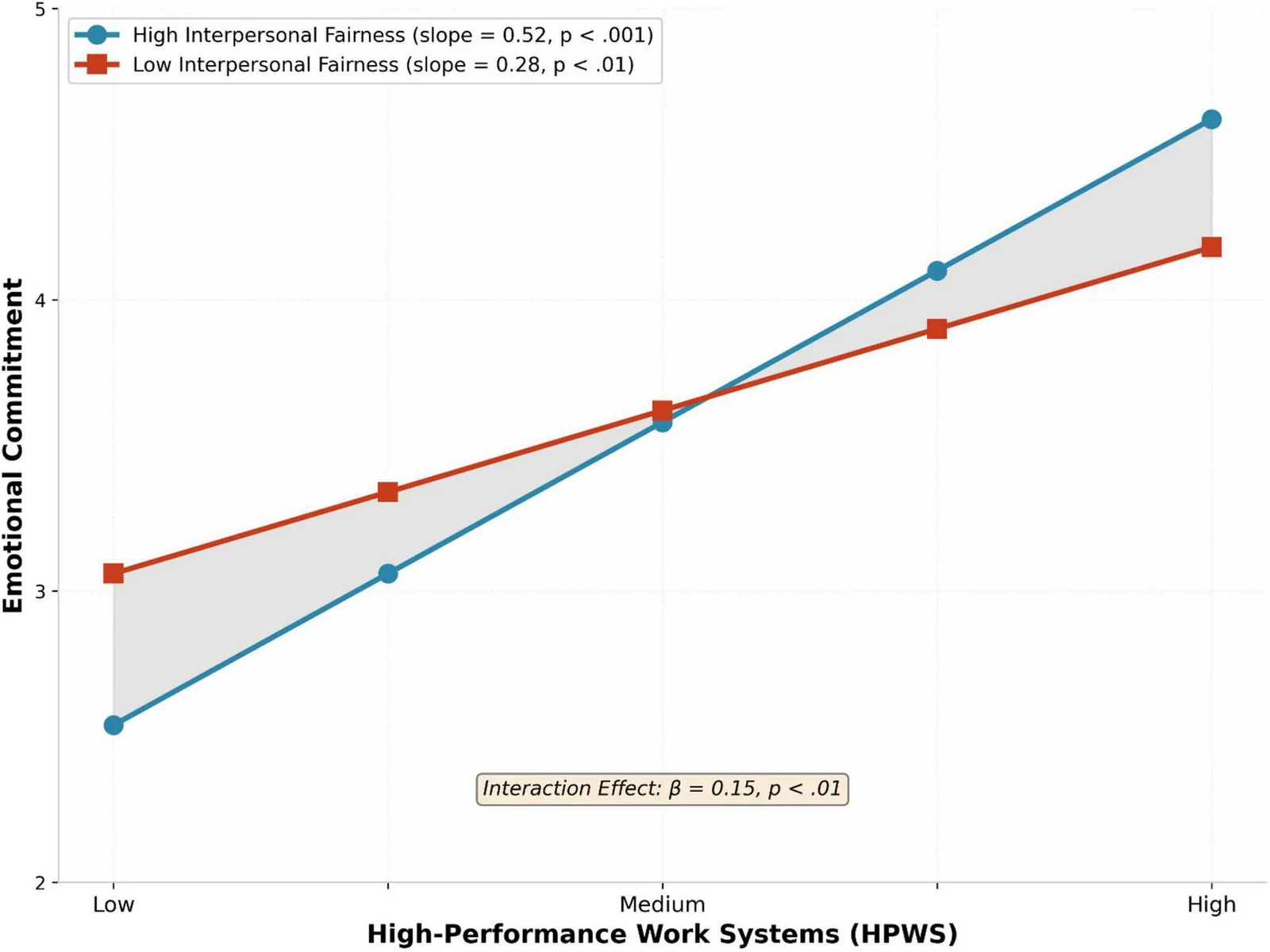
The moderating effect of interpersonal fairness on the HPWS-emotional commitment relationship.

Hypothesis 3 predicted that interpersonal fairness moderates the relationship between HPWS and emotional commitment. The results from Model 4 in Table 2 show that the interaction between HPWS and interpersonal fairness was significant and positive (beta = 0.15, *p* < 0.01), supporting Hypothesis 3. Simple slope analysis (Figure 2) revealed that the relationship between HPWS and emotional commitment was stronger when interpersonal fairness was high (simple slope = 0.52, *p* < 0.001) than when it was low (simple slope = 0.28, *p* < 0.01).

Hypothesis 4 proposed that interpersonal fairness moderates the indirect effect of HPWS on subjective well-being through emotional commitment. The moderated mediation analysis showed that the index of moderated mediation was significant (index = 0.06, 95% confidence interval [0.02, 0.11]), supporting Hypothesis 4. Specifically, the indirect effect was stronger when interpersonal fairness was high (indirect effect = 0.22, 95% confidence interval [0.15, 0.30]) than when it was low (indirect effect = 0.12, 95% confidence interval [0.06, 0.19]). Model 5 also provides indirect evidence for this argument.

### 4.4 Additional analyses

We conducted several additional analyses to ensure the robustness of our findings. First, we tested for potential nonlinear effects of HPWS on subjective well-being by including a quadratic term in the regression equation. The quadratic term was not significant (beta = −0.03, *p* = 0.42), suggesting that the relationship is linear rather than curvilinear. Second, we examined whether the results varied across demographic groups by including interaction terms between HPWS and gender, age, and education level; none was significant, indicating that the effects of HPWS on subjective well-being were consistent across demographic groups. Third, we tested an alternative model in which subjective well-being mediates the relationship between HPWS and emotional commitment; this model fit the data more poorly than our hypothesized model, supporting the proposed causal ordering. Fourth, we re-estimated all models without the demographic control variables; the pattern and statistical significance of the hypothesized effects were unchanged, indicating that the results are not artifacts of the control set. Fifth, we repeated the analyses excluding teams with fewer than three responding members to check the influence of small teams; the results were materially identical.

## 5 Discussion

### 5.1 General discussion

In general terms, our results show that high-performance work systems can be good for people. Employees in teams with more fully implemented HPWS reported higher subjective well-being, and a substantial share of this benefit flowed through a warmer psychological relationship with the organization: employees felt more emotionally committed, and this commitment translated into more positive evaluations of their lives. Just as importantly, the benefit was not automatic. It materialized fully only when supervisors treated employees with dignity and respect; where interpersonal fairness was low, the well-being dividend of HPWS was substantially reduced. In short, what organizations provide through HPWS matters, but how managers treat employees while providing it matters just as much.

These findings speak to each hypothesis and to the prior literature. Hypothesis 1 was supported: HPWS related positively to subjective well-being. This aligns with the mutual gains perspective and with meta-analytic and review evidence that HR investment can benefit employees as well as employers (Jiang et al., 2012; Peccei and Van De Voorde, 2019), but it diverges from studies reporting strain-based costs of high-performance practices (Guest, 2017; Katou, 2022). The positive main effect also converges with recent evidence from hospitality and other high-intensity service settings, where HPWS improved satisfaction and reduced burnout (Dorta-Afonso et al., 2023). Our need-based framing reconciles the discrepancy: the direction of the HPWS effect depends on the need-supportive quality of enactment, which our design explicitly modeled rather than assumed away. Hypothesis 2 was supported: emotional commitment partially mediated the HPWS-well-being link. This extends attribution research, which established that employees’ interpretations of HR practices drive their well-being reactions (Van De Voorde and Beijer, 2015), by specifying the internalization mechanism that produces those interpretations (Gagne and Deci, 2005; Deci et al., 2017); it also resonates with evidence that affective commitment covaries with well-being (Meyer and Maltin, 2010; Alothmany et al., 2023). Hypothesis 3 was supported: interpersonal fairness strengthened the HPWS-commitment link, consistent with meta-analytic evidence that interactional justice shapes employee responses to managerial action (Colquitt et al., 2013), while our need-support account explains why this contingency arises. Hypothesis 4 was supported: the indirect effect was conditional on interpersonal fairness along the whole causal chain, corroborating the moderated mediation logic (Hayes, 2015) and contradicting the implicit assumption in much HPWS research that implementation quality is background noise rather than a constitutive condition of effectiveness.

### 5.2 Theoretical contributions

We position this study as an exercise in context-sensitive theory development of the kind advocated by Homer and Lim (2024): combining doing as the Romans do – documenting how HPWS actually operates in China’s technology sector – with understanding why the Romans do it – explaining the motivational process that makes HPWS succeed or fail in that setting. Because the study is national and industry-specific, its contribution is best read at the meso level: not a claim of universal law, but a specification of how a general theory operates within a defined scope, and of the scope conditions under which its predictions should travel.

Within that scope, the study develops self-determination theory in several interconnected ways. It extends SDT’s contextual reach into strategic human resource management: although SDT is among the most influential theories of work motivation, its application to system-level HR architectures has been limited (Deci et al., 2017; Gagne et al., 2022), and our demonstration that the HPWS-well-being relationship follows basic-need logic in a high-pressure, non-Western context supports the portability of SDT’s core propositions while making the tested scope conditions explicit (Homer and Lim, 2024). The study also supplies the psychological foundation that HPWS research has lacked: by theorizing and testing the chain from need satisfaction through internalization and attachment to well-being, it converts emotional commitment from a convenient mediator into a theory-derived mechanism and thereby explains, rather than merely documents, employees’ cognitive and affective interpretation of HPWS (Van De Voorde and Beijer, 2015). Perhaps most importantly, the study reframes the long-standing mutual gains versus conflicting outcomes debate: on our account, the two camps are not contradictory but conditional, because HPWS tends to benefit well-being when enacted in a need-supportive interpersonal climate and to disappoint when enacted in a controlling one. This constitutes substantive theoretical integration – both camps emerge as special cases of one motivational process – rather than a parallel listing of perspectives. Finally, by specifying interpersonal fairness as an enabling rather than buffering moderator, the study clarifies the nature of the moderation effect and connects the organizational justice literature to motivation theory, opening a precise agenda for future work on the interactional conditions of HR effectiveness (Colquitt et al., 2013). In doing so, the study also answers recent calls to disentangle the resource- and demand-side pathways through which HR practices affect well-being (Li et al., 2024) and positions SDT alongside complementary frameworks such as job demands-resources theory (Demerouti and Bakker, 2023).

### 5.3 Practical recommendations

For practitioners, the central message is that the return on HPWS investment is delivered – or destroyed – at the point of interpersonal enactment. Organizations can act on this insight in concrete ways. When designing HPWS, they can involve employees in co-designing goals, appraisal criteria, and involvement mechanisms, because co-design converts practices done to employees into practices owned by employees and directly supports autonomy. When implementing performance management, they can train supervisors with behaviorally specific protocols – for example, structured feedback conversations that begin with the employee’s self-assessment, separate developmental from evaluative components, and end with jointly owned action plans – and assess that training through behavioral rehearsal rather than attendance. When rolling out new HR practices, they can run a need-support check: brief pulse surveys measuring whether employees experience the practice as supporting their autonomy, competence, and relatedness, with the results reviewed before the practice is scaled. More broadly, organizations can treat emotional commitment as a leading indicator of well-being in their people-analytics dashboards, so that eroding attachment triggers intervention before well-being and performance decline; use 360-degree feedback on supervisors’ interpersonal conduct and make respectful treatment an explicit criterion in managers’ own evaluations and promotion decisions; and pair demanding performance goals with recovery resources – protected offline time, realistic staffing, and workload monitoring – so that challenge does not tip into need thwarting. We also recognize an implementation limit: these actions require sustained investment in first-line management capability, and in cost-cutting periods such investment is often the first to be sacrificed – precisely when its protective value is greatest.

### 5.4 Policy recommendations

At the organizational-policy level, our findings argue for institutionalizing well-being governance. Boards and executive committees can adopt employee well-being metrics alongside financial key performance indicators, require HR audits that assess not only the presence of HPWS practices but also the quality of their enactment, and embed interpersonal-fairness standards into leadership competency models and promotion criteria, so that need-supportive management becomes a matter of policy rather than personal style. At the governmental level, the findings are relevant to the ongoing regulation of work intensity in China’s technology sector. The 2021 joint guidance of the Supreme People’s Court and the Ministry of Human Resources and Social Security on statutory working-hour limits addresses the demand side of the well-being problem (Supreme People’s Court and Ministry of Human Resources and Social Security of the People’s Republic of China, 2021); our results suggest complementing it with the supply side – policies that raise the quality of everyday management. Examples include publicly funded supervisor-training and certification programs in interpersonal and feedback skills; the extension of existing model-employer and labor-standard certification schemes to include employee well-being indicators, with preferential treatment for certified firms; and industry-level occupational mental-health standards for high-intensity sectors. Such policies would amplify the private returns our study identifies: firms that implement HPWS in a need-supportive way gain healthier and more committed employees, while the wider economy gains a more sustainable basis for innovation-led growth.

### 5.5 Limitations and future research directions

This study has several limitations that should be acknowledged. First, although we employed a time-lagged research design to establish temporal precedence, the data are still correlational in nature, which limits our ability to make definitive causal inferences. Future research could employ experimental or quasi-experimental designs to provide stronger evidence of causality. Moreover, although we combined a time-lagged, multi-source design with procedural and statistical checks, common method bias cannot be entirely ruled out for the employee-reported variables; recent methodological work shows that such bias is complex, widespread, and not amenable to any single remedy (Podsakoff et al., 2024; Howard et al., 2024).

Second, our sample was drawn from technology companies in China, which may limit the generalizability of our findings to other industries and cultural contexts. Future research should replicate our findings in different industries and countries to assess the robustness of our results across diverse settings.

Third, we focused on emotional commitment as a mediator and interpersonal fairness as a moderator, but other mechanisms and boundary conditions may also be relevant. Future research could explore additional mediators such as job satisfaction, work engagement, or psychological empowerment, as well as additional moderators such as organizational culture, leadership style, or individual differences.

Fourth, we measured HPWS at the team level based on team leaders’ reports, which may not fully capture employees’ individual perceptions of HPWS. Future research could examine how individual perceptions of HPWS differ from team-level assessments and how these different levels of analysis influence employee outcomes.

Finally, our study focused on subjective well-being as the outcome variable, but HPWS may also influence other aspects of employee well-being, such as physical health, psychological distress, or work-life balance. Future research could adopt a more comprehensive approach to employee well-being by examining multiple dimensions of well-being simultaneously.

In addition, future research could exploit self-determination theory more fully than we were able to. We inferred need satisfaction and internalization from the pattern of results rather than measuring them directly; incorporating validated measures of basic need satisfaction and need thwarting as mediators between HPWS and emotional commitment would provide a sharper test of the proposed mechanism (Van den Broeck et al., 2016). Experience-sampling and longitudinal designs could trace how individual episodes of fair or unfair interpersonal treatment accumulate into team climates, and cross-cultural replications could test the universality of the need-support logic across institutional contexts (Gagne et al., 2022; Homer and Lim, 2024). Integrating self-determination theory with job demands-resources theory could further clarify how HPWS operates simultaneously as a resource and a demand, and how boundary conditions such as interpersonal fairness shift that balance (Demerouti and Bakker, 2023).

## 6 Conclusion

This study set out to explain how and when high-performance work systems enhance employees’ subjective well-being. Drawing on self-determination theory and three-wave, multi-source data from 412 employees in 68 Chinese technology teams, we found that HPWS improves well-being partly by strengthening emotional commitment – the affective bond that forms when HR practices satisfy employees’ needs for autonomy, competence, and relatedness – and that this pathway depends on interpersonal fairness: the well-being benefits of HPWS are realized when, and only when, practices are enacted with dignity and respect.

The broader lesson is that performance systems and human well-being are not inherently in conflict. When organizations design HPWS around basic psychological needs and equip supervisors to deliver them in a need-supportive manner, high performance and high well-being can be pursued as joint rather than competing goals. We hope this need-based account helps move the HPWS-well-being debate beyond the question of whether these systems affect employees toward the more useful question of how organizations can ensure that they affect employees for the better.

## Data Availability

The datasets presented in this article are not readily available because it is belong to the secret of my organization. Requests to access the datasets should be directed to RL, rucck@163.com.
